# Complement factors C3a and C5a mimick a proinflammatory microenvironment and increase HBV IGRA sensitivity

**DOI:** 10.1186/s12967-018-1752-8

**Published:** 2019-01-03

**Authors:** Katharina Bröker, Robin Terzenbach, Frank Bentzien, Stefan Lüth, Werner Dammermann

**Affiliations:** 1 Center of Internal Medicine II, Brandenburg Medical School, University Hospital Brandenburg, Hochstrasse 29, 14770 Brandenburg, Germany; 2 Department of Anatomy and Experimental Morphology, University Medical Center Hamburg-Eppendorf, Martinistrasse 52, 20246 Hamburg, Germany; 3 Department of Transfusion Medicine, University Medical Center Hamburg-Eppendorf, Martinistrasse 52, 20246 Hamburg, Germany

**Keywords:** HBV, CRA, IGRA, Complement, Anaphylatoxins, C3a, C5a

## Abstract

**Background:**

Hepatitis B virus (HBV) infections represent a global health problem and chronic hepatitis B (CHB) leads to liver cirrhosis and hepatocellular carcinoma. Thus, timely diagnosis of hepatitis B is crucial to ensure adequate treatment. We developed a powerful and rapid whole blood-based cytokine release assay assessing cellular immune responses to HBV antigens. IL-2 and IFNγ release in this assay depicts hepatitis B vaccination status. Of note, CHB goes along with elevated C5a concentrations in plasma. We aim at mimicking the proinflammatory microenvironment associated with HBV infection to enhance the diagnostic quality of our HBV specific cytokine release assay. We specifically investigated the potential of the complement factors C3a and C5a as costimulators and analyzed their potential effects on activation marker expression on T cells and antigen presenting cells.

**Results:**

Whole blood from 87 healthy individuals (n = 59 hepatitis B vaccinated, n = 28 unvaccinated) was stimulated with HBV surface antigen (HBsAg) in presence or absence of C3a or C5a, respectively. Further, C3a and C5a were used in combination to investigate potential synergistic effects. IL-2 and IFNγ levels in plasma were quantified using ELISA. Complement factor C5a specifically enhances HBsAg-mediated IL-2 (690.3 ± 195.4 pg/ml vs. 789.4 ± 216.5 pg/ml) and IFNγ (146.0 ± 43.1 pg/ml vs. 336.7 ± 67.9 pg/ml) responses in whole blood. Similar cytokine levels were measured when both C3a and C5a were used. With a diagnostic specificity of 90% the IFNγ release assay reached a diagnostic sensitivity of 49.2% upon whole blood stimulation with HBsAg alone, but of 78.9% when HBsAg was combined with C3a and C5a.

**Conclusions:**

Innate signals mediated via complement pathways contribute to HBV-specific cellular immune responses. The massively improved diagnostic sensitivity of the IFNγ release assay after addition of C3a and C5a demonstrates that these effects render whole blood-based cytokine release assays even more potent as screening tools in HBV immunology and HBV vaccination studies.

**Electronic supplementary material:**

The online version of this article (10.1186/s12967-018-1752-8) contains supplementary material, which is available to authorized users.

## Background

HBV is a small enveloped DNA virus belonging to the *Hepadnaviridae* family of viruses. The virus is transmitted by sexual, parenteral and vertical route. Acute hepatitis B infections often become chronic and more than 240 million individuals worldwide suffer from chronic hepatitis B. These patients are at high risk to develop severe liver disease, liver cirrhosis or hepatocellular carcinoma [1]. In order to start an adequate treatment and to take measures to prevent virus transmission, a timely diagnosis is of paramount importance.

Humoral immunity can easily be assessed using ELISA, whereas methods addressing cell-mediated immunity are still quite challenging [2]. Thanks to their easy handling and robustness whole blood-based cytokine release assays represent an attractive alternative. Briefly, whole blood is incubated with distinct antigens whereupon cell-mediated immunity is directly assessed quantifying the amount of memory T cell-derived cytokines in plasma.

HBV carries different antigens including the hepatitis B surface antigen (HBsAg) as part of the virus envelope, which is also a major component of the available prophylactic HBV vaccine as well as the hepatitis B core antigen (HBcAg) as part of the capsule.

As published previously, we developed a whole blood-based cytokine release assay capable of assessing T cell responsiveness to the HBV antigens HBsAg and HBcAg. This assay allows reliable discrimination between hepatitis B patients, vaccinated and unvaccinated healthy individuals [3]. Unfortunately, due to the rather low amount of antigen-specific memory T cells in whole blood, the levels of antigen-induced cytokine responses are accordingly low. In line with previous studies, we found that diagnostic sensitivity of those cytokine release assays can be improved by activation of toll like receptors (TLRs) which enhance antigen-induced cytokine production [4–6].

The complement system is a central part of the innate immune system and comprises a variety of different plasma proteins. Activation of this danger sensing system results in a proteolytic cascade. In line with this, the peptides C3a and C5a are generated. These so-called anaphylatoxins are small peptide fragments which exert different, mostly proinflammatory effector functions, by binding to their cognate receptors. Multiple studies could demonstrate that the anaphylatoxins modulate cytokine responses in general [7–9]. Of specific interest for our test system is data demonstrating that the anaphylatoxin C5a is capable of regulating the IFNγ production of human CD4^+^ T cells [8]. Moreover, patients with chronic hepatitis B show elevated C5a concentrations in plasma and these C5a levels even positively correlate with clinical parameters reflecting disease severity [10]. All this renders the anaphylatoxins C3a and C5a interesting candidates for application in our cytokine release assay.

In the current study, we aimed at increasing the diagnostic sensitivity and specificity of the HBV-specific IL-2 and IFNγ release assay using the complement factors C3a and C5a as costimulators thereby mimicking the proinflammatory microenvironment in HBV patients in vivo. Indeed, we could show that stimulation of whole blood with the anaphylatoxin C5a enhanced HBsAg-specific IL-2 and IFNγ production. Our data further demonstrate that both C3a and C5a are capable of increasing the sensitivity of the IFNγ release assay by up to 29.8% (49.2% → 78.9%).

## Results

### C5a enhances HBsAg-specific IFNγ and IL-2 responses in whole blood of healthy donors

**Fig. 1:**
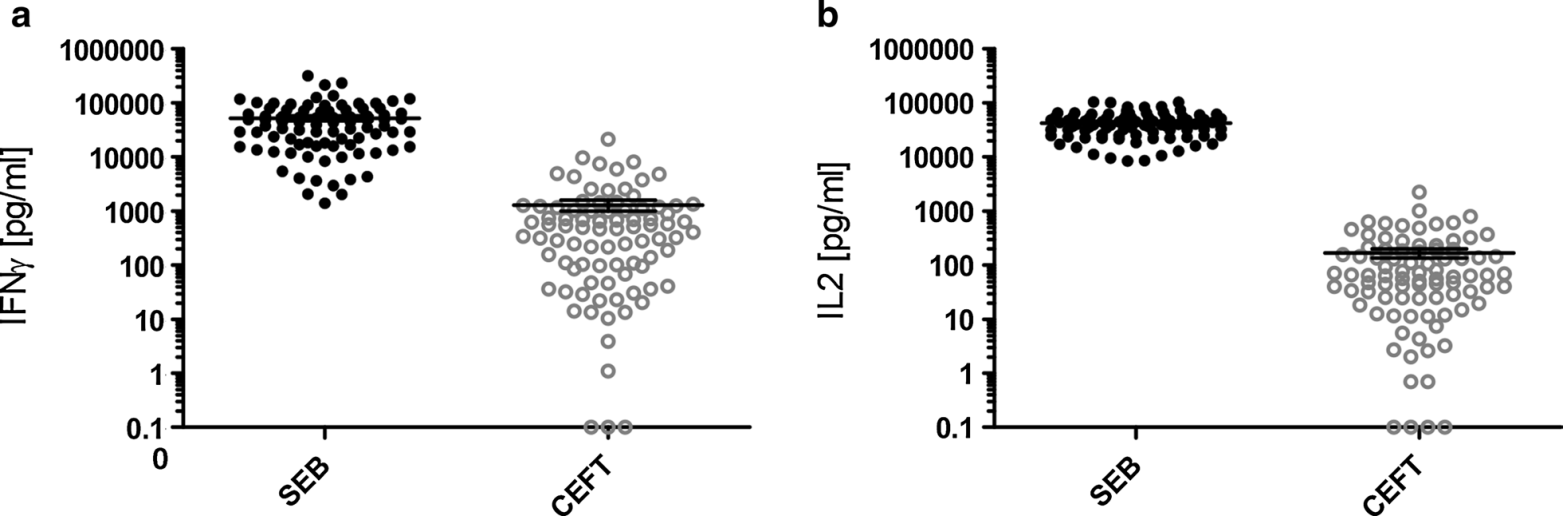
Whole blood of healthy donors responds to SEB and CEFT positive control stimulation. **a** IFNγ and **b** IL2 secretion in whole blood upon stimulation with SEB and CEFT, respectively. All depicted values are corrected by the negative control and are given as mean concentration in pg/ml ± SEM. Lower limit of detection (Background + 3 × SD) was at 1.9–12.6 pg/ml for IFNγ and at 2.6–9.8 pg/ml for IL2 (n = 87)

As a first step, cell viability in all collected samples was assessed by stimulating whole blood with SEB and CEFT, respectively. All samples showed strong IFNγ (51,922 ± 5527 pg/ml) as well as IL-2 (42,295 ± 2280 pg/ml) responses to SEB and prominent, but in comparison much weaker cytokine secretion upon CEFT stimulation (1294 ± 302.8 pg/ml IFNγ and 167.5 ± 32.01 pg/ml IL-2) (Fig. 1).

**Fig. 2:**
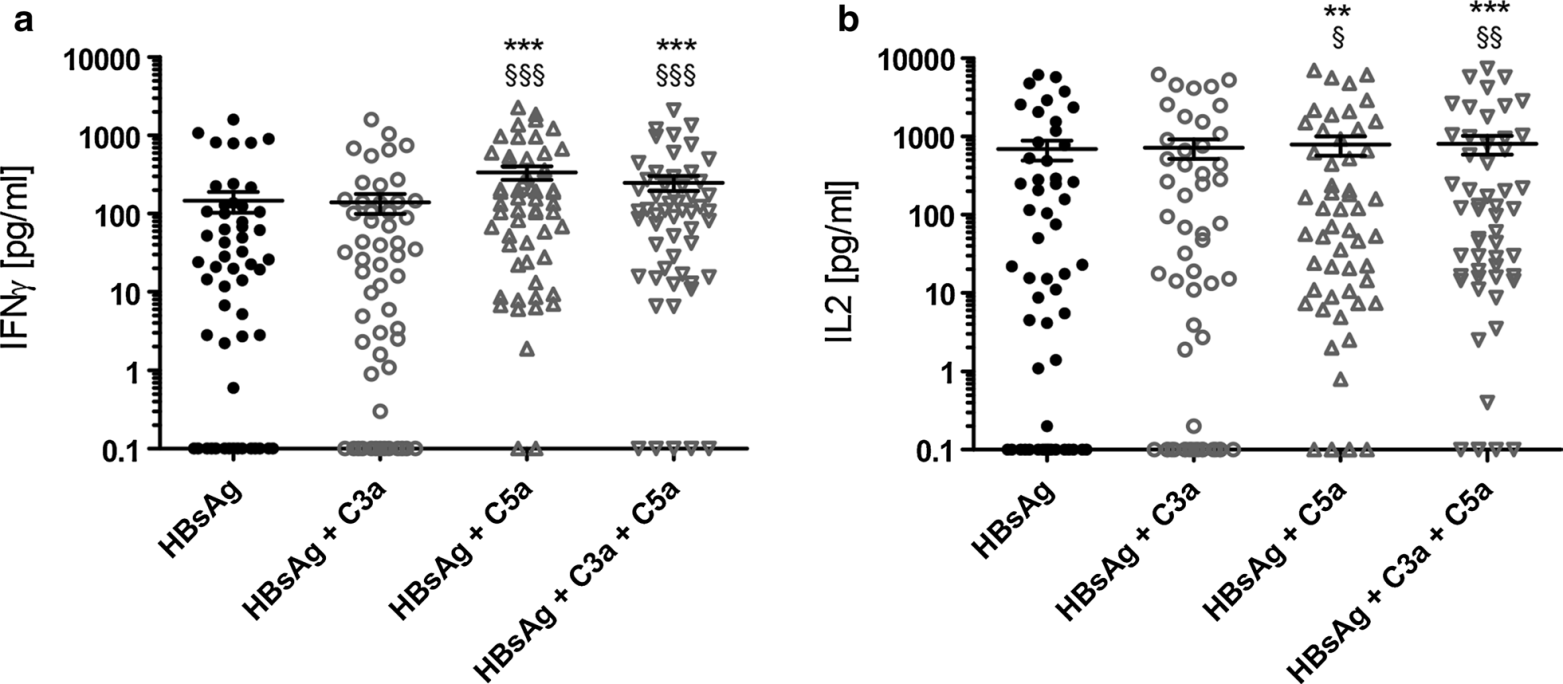
C5a specifically enhances IFNγ and IL2 responses to HBsAg in whole blood of healthy donors. **a** IFNγ and **b** IL2 responses in whole blood following stimulation with HBsAg, HBsAg and C3a, HBsAg and C5a as well as HBsAg, C3a and C5a. All depicted values were corrected by the negative control and are given as mean concentration pg/ml ± SEM. Lower limit of detection (Background + 3 × SD) was at 1.9–12.6 pg/ml for IFNγ and at 2.6–9.8 pg/ml for IL2. Shapiro–Wilk normality test followed by Friedman test. Following symbols pinpoint significant differences: *, ^§^One symbol equals p < 0.05, two symbols p < 0.01, three symbols p < 0.001 (n = 55)

Stimulation of whole blood with synthetic HBsAg peptides induced production of both, IFNγ (146 ± 43.1 pg/ml) and IL-2 (690.3 ± 195.4 pg/ml) with the cytokine responses for IL-2 (Fig. 2b) being generally stronger than the ones for IFNγ (Fig. 2a).

Additional stimulation with C3a did not have any significant effect on the release of the mentioned cytokines (139 ± 39.6 pg/ml IFNγ and 719.6 ± 198.9 pg/ml IL-2) whereas combination of HBsAg with C5a significantly increased the HBsAg-specific production of IFNγ (336.7 ± 67.9 pg/ml, p < 0.0001) and IL-2 (789.4 ± 216.5 pg/ml, p < 0.0001) (Fig. 2).

### C3a and C5a do not show a positive synergistic effect on HBsAg-specific IFNγ and IL-2 responses in whole blood of healthy donors

To test if C3a and C5a synergize to further increase HBsAg-induced IFNγ and IL-2 production, whole blood was stimulated with HBsAg, C3a and C5a. Even though samples stimulated with HBsAg, C3a and C5a showed a significantly stronger cytokine production than samples stimulated with HBsAg alone, combination of both complement fragments did not lead to a significant increase of IFNγ (248.2 ± 53.8 pg/ml) (Fig. 2a) and IL-2 (803.7 ± 217.9 pg/ml) (Fig. 2b) secretion when compared to samples stimulated with HBsAg and C5a alone. Anyhow, samples stimulated with HBsAg, C3a and C5a showed stronger IFNγ (248.2 ± 53.8 pg/ml) (Fig. 2a) as well as IL-2 responses (803.7 ± 219.9 pg/ml) (Fig. 2b) than samples stimulated with HBsAg and C3a alone (IFNγ 139 ± 39.6 pg/ml, IL-2 719.6 ± 198.9 pg/ml).

Interestingly, addition of C3a seems to slightly decrease the amount of secreted IFNγ since the cytokine concentration was lower after stimulation with HBsAg, C3a and C5a than after stimulation with HBsAg and C5a alone (336.7 ± 67.9 pg/ml after stimulation with HBsAg + C5a and 248.2 ± 53.8 pg/ml after stimulation with HBsAg + C3a + C5a), but showed an opposite effect with regard to IL-2 (789.4 ± 215.5 pg/ml after stimulation with HBsAg + C5a and 803.7 ± 219.9 pg/ml after stimulation with HBsAg + C3a + C5a).

### Additional stimulation of whole blood with the complement components C3a and C5a improves IFNγ release assay sensitivity

**Fig. 3:**
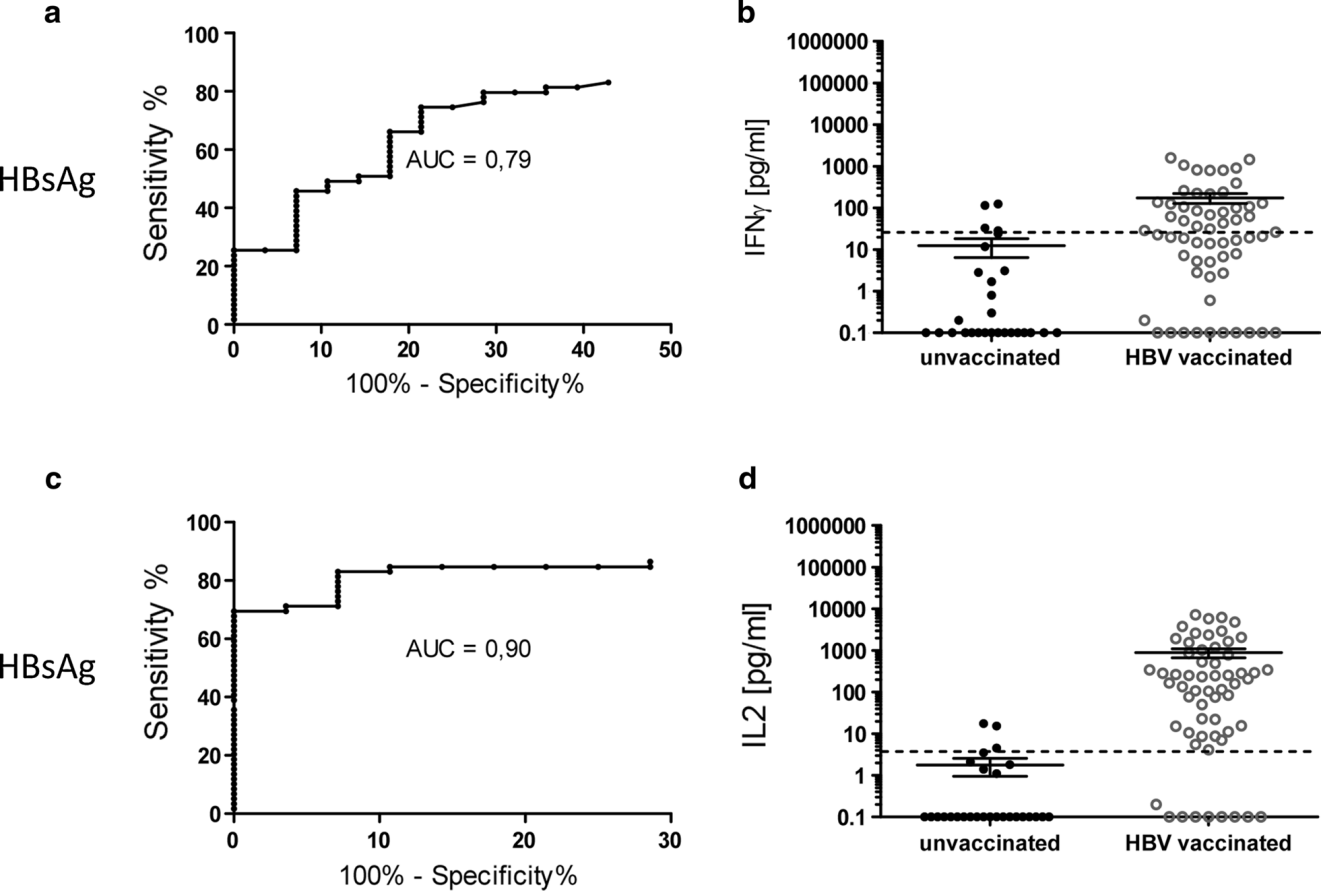
HBsAg-specific IFNγ and IL2 production depict the hepatitis B vaccination status. **a** IFNγ and **b** IL2 responses were assessed upon stimulation of whole blood with HBsAg. Shown are for each cytokine **a**, **c** receiver-operating-characteristic (ROC) curves including calculated AUC (area under the curve) as well as **b**, **d** scatter plot of cytokine release of HepB vaccinated and unvaccinated HC after stimulation of whole blood. All depicted values were corrected by the negative control. Dotted line indicates **b** IFNγ cut-off at 28.45 pg/ml and **d** IL2 cut-off at 3.8 pg/ml (n = 87; n = 28 anti-HBs negative and n = 59 anti-HBs positive)

Since HBsAg is a main component of the prophylactic hepatitis B vaccine it does not surprise that the HBsAg-mediated production of the cytokines IL-2 and IFNγ correlates with the blood donors’ vaccination status as published previously [3, 6]. Within our study cohort, 59/87 individuals showed positive anti-HBs antibody titers whereas 28/87 individuals were anti-HBs negative (Table 1). In accordance with our preexisting data successful vaccination was in most cases linked to cytokine levels crossing a certain threshold as calculated by ROC analysis. With regard to IFNγ secretion, 29/59 values measured for anti-HBs positive blood donors lay above the calculated threshold of 28.5 pg/ml in response to stimulation with HBsAg (Fig. 3b). ROC analysis further revealed that at a diagnostic specificity of 90% the according assay reached a diagnostic sensitivity of only 49.2% (Fig. 3a).

**Table 1:** Characteristics of all blood donors included in the study

Characteristic	Healthy donors, hepatitis B vaccinated	Healthy donors, unvaccinated
n	59 (67.8%)	28 (32.2%)
Male	37 (62.7%)	17 (60.7%)
Female	21 (35.6%)	10 (35.7%)
nd	1 (1.7%)	1 (3.6%)
Age (year)^a^	40.08 ± 14.15	53.85 ± 9.7
Male	42.54 ± 14.69	54.76 ± 11.66
Female	35.73 ± 12.28	52.30 ± 5.06
nd^b^	–	–

^a^The data are shown as means ± standard deviations

^b^For one hepatitis B vaccinated and one unvaccinated donor neither gender nor age were provided

The correlation between the blood donors’ vaccination status and the HBsAg-driven cytokine response was more pronounced with regard to IL2 secretion. In this case, 50/59 vaccinated blood donors crossed the calculated threshold of 3.8 pg/ml when their blood was stimulated and IL2 levels were measured (Fig. 3d). In comparison to the IFNγ release assay the IL2 release assay already reached a diagnostic sensitivity of 84.75% at a diagnostic specificity of 90%, even without combination of HBsAg with additional stimuli (Fig. 3c).

**Fig. 4:**
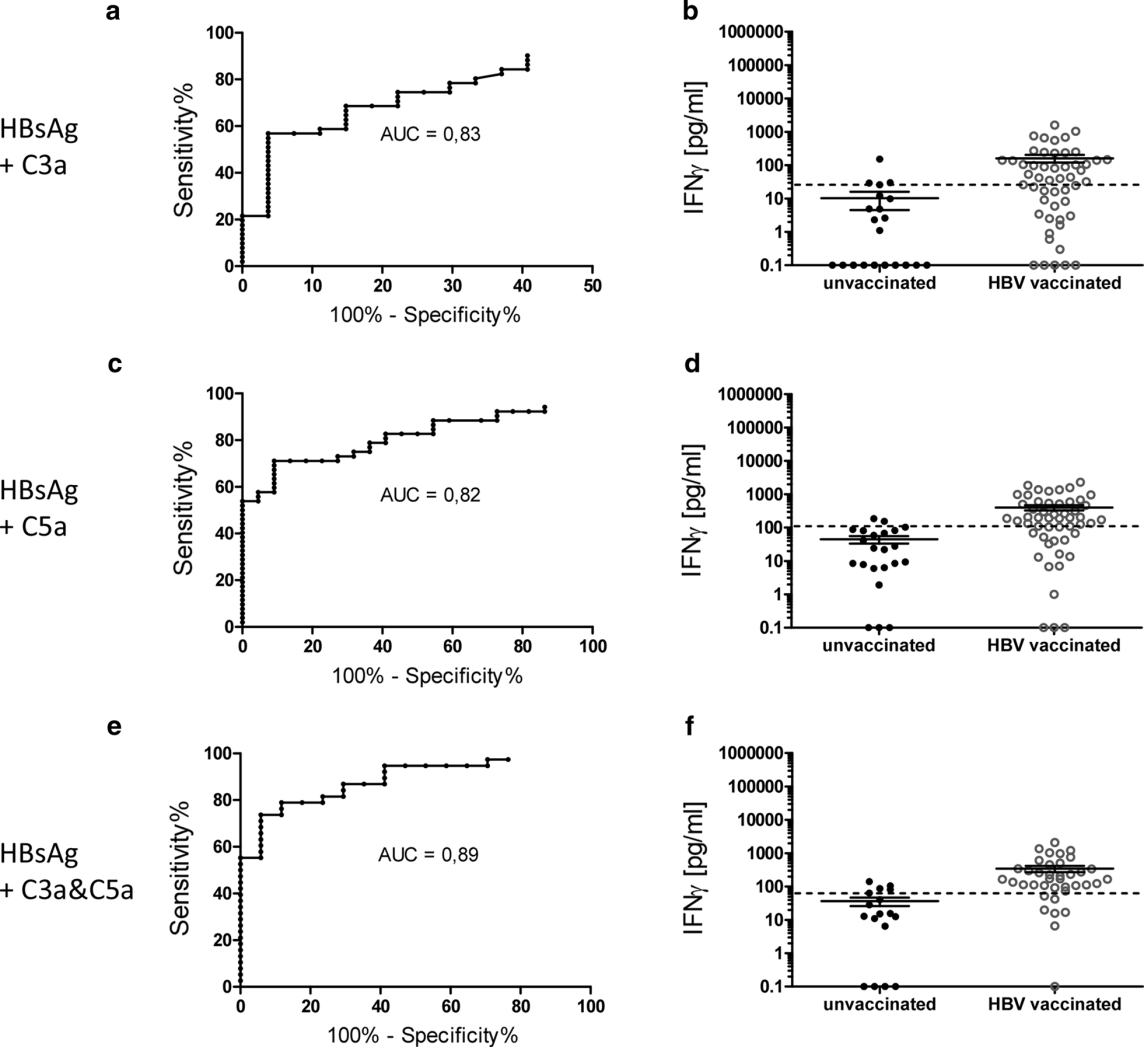
Additional stimulation of whole blood with C3a and C5a improves IFNγ release assay sensitivity. IFNγ responses to HBsAg were assessed in presence of **a**, **b** C3a, **c**, **d** C5a as well as **e**, **f** C3a and C5a. Shown are for each condition **a**, **c**, **e** receiver-operating-characteristic (ROC) curves including calculated AUC (area under the curve) as well as **b**, **d**, **f** scatter plot of IFNγ release of HepB vaccinated and unvaccinated HC after stimulation of whole blood. All depicted values were corrected by the negative control. Dotted line indicates IFNγ cut-off at **b** 25.95 pg/ml (n = 78; n = 27 anti-HBs positive and n = 51 anti-HBs negative), **d** 103.5 pg/ml (n = 74; n = 22 anti-HBs positive and n = 52 anti-HBs negative), **f** 88.6 pg/ml (n = 55; n = 17 anti-HBs negative and n = 38 anti-HBs positive)

Anyhow, combination of HBsAg with the complement factors C3a and C5a positively affected the diagnostic sensitivity of the IFNγ release assay. Upon stimulation with HBsAg and C3a the diagnostic sensitivity of the assay reached 56.9% (Fig. 4a), 71.2% when HBsAg was combined with C5a (Fig. 4c) and even 78.9% when blood was stimulated with HBsAg and both complement fragments (Fig. 4e).

In line with that, the corresponding AUC value increased from 0.79 to 0.89 (Figs. 3a, c, 4a, c, e).

**Fig. 5:**
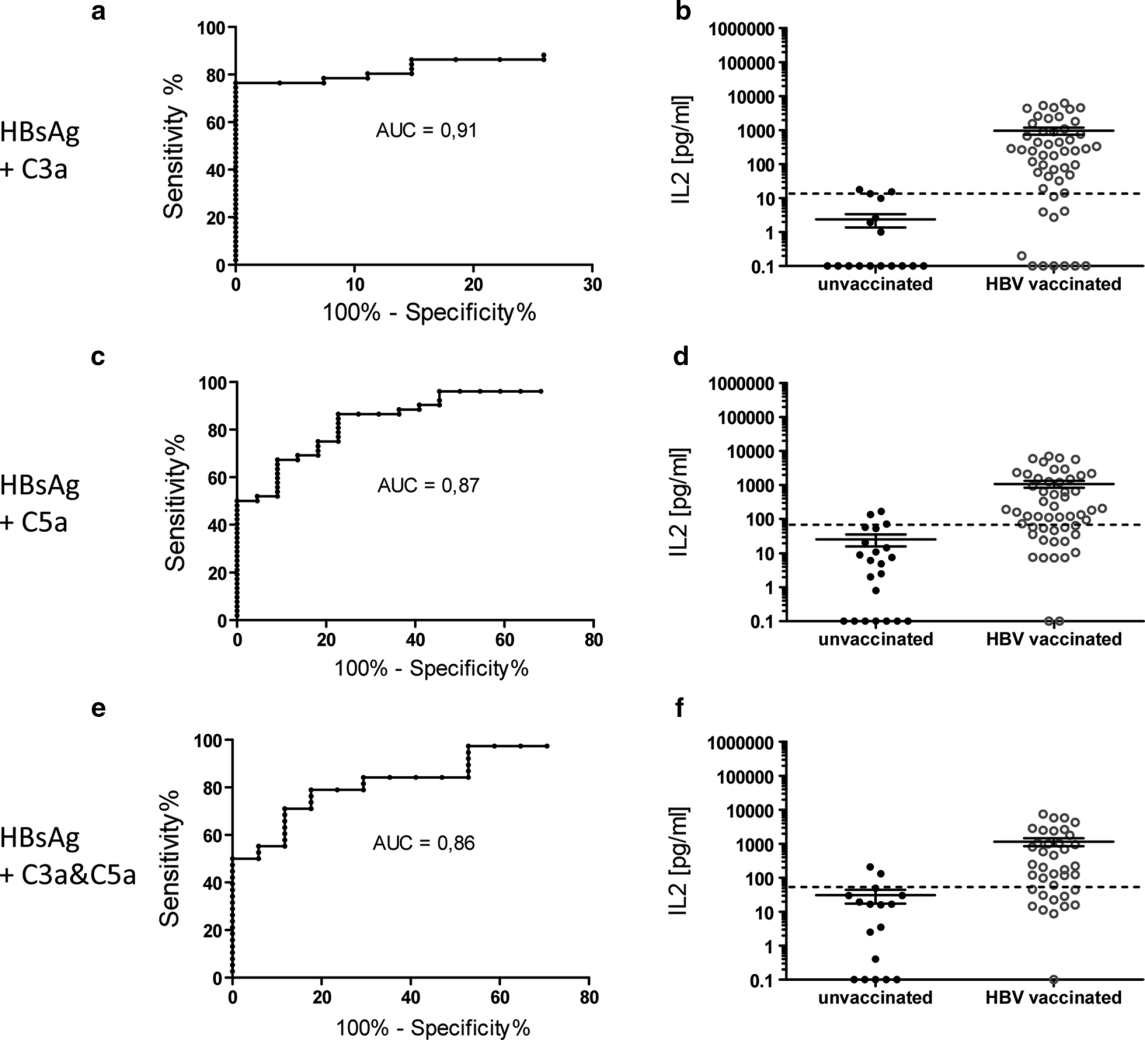
Additional stimulation of whole blood with with C3a and C5a does not affect IL2 release assay sensitivity. IL2 responses to HBsAg were assessed in presence of **a**, **b** C3a, **c**, **d** C5a as well as **e**, **f** C3a and C5a. Shown are for each condition **a**, **c**, **e** receiver-operating-characteristic (ROC) curves including calculated AUC (area under the curve) as well as **b**, **d**, **f** scatter plot of IL2 release of HepB vaccinated and unvaccinated HC after stimulation of whole blood. All depicted values were corrected by the negative control. Dotted line indicates IL2 cut-off at **b** 10.4 pg/ml (n = 78; n = 27 anti-HBs positive and n = 51 anti-HBs negative), **d** 71.7 pg/ml (n = 74; n = 22 anti-HBs positive and n = 52 anti-HBs negative), **f** 55 pg/ml (n = 55; n = 17 anti-HBs negative and n = 38 anti-HBs positive)

With regard to the IL2 release assay neither C3a nor C5a did have any positive effect on the diagnostic sensitivity of this assay (Fig. 5a, c, e).

### Anti-HBs titers correlate with strength of the cytokine response

**Fig. 6:**
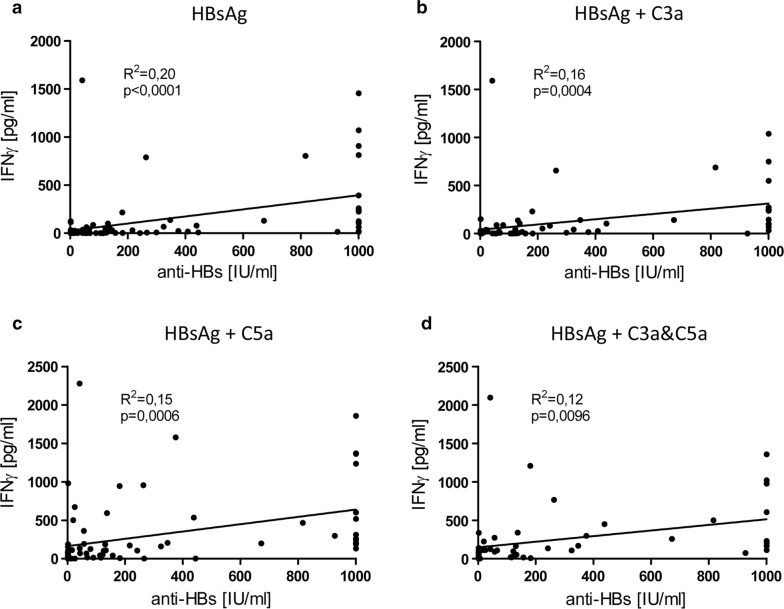
Anti-HBs titers and strength of IFNγ response correlate in hepatitis B vaccinated HC. Scatter plot of IFNγ release of HepB vaccinated HC vs. anti-HBs titers after stimulation of whole blood with **a** HBsAg alone (n = 87), **b** HBsAg and C3a (n = 80), **c** HBsAg and C5a (n = 74) as well as **d** HBsAg, C3a and C5a (n = 55). Antigen induced cytokine responses were corrected by the negative control values. Continuous line indicates linear regression and Pearson correlation. R^2^: coefficient of determination

In the present study we could further show a clear correlation between the amount of secreted cytokines and the measured anti-HBs antibody. Thus, there was a link between the anti-HBs titer and the strength of the IFNγ response, no matter if the blood was stimulated with HBsAg alone (Fig. 6a, R^2^ = 0.20 and p < 0.0001) or with HBsAg and complement fragments (Fig. 6, (b) R^2^ = 0.16 and p = 0.0004 for HBsAg + C3a stimulation; (c) R^2^ = 0.15 and p = 0.0006 for HBsAg + C5a stimulation; (d) R^2^ = 0.12 and p = 0.0096 for HBsAg + C3a and C5a stimulation).

**Fig. 7:**
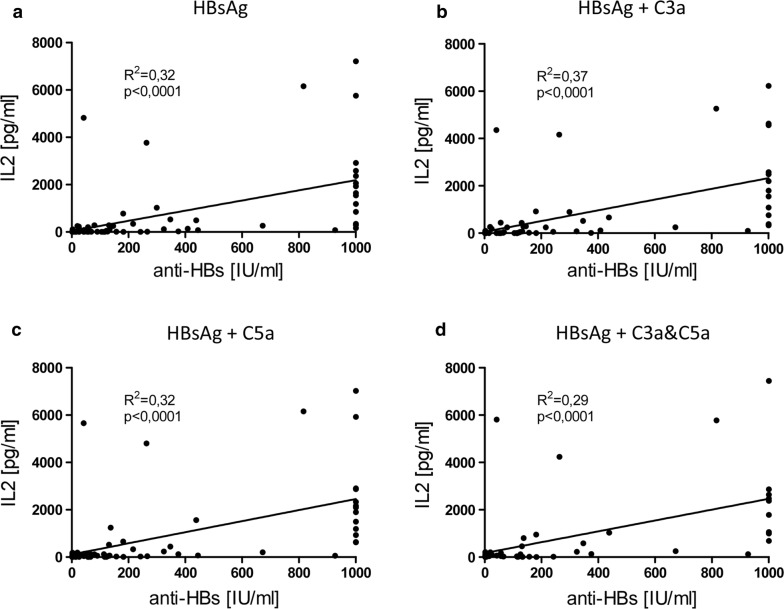
Anti-HBs titers and strength of IL2 response correlate in hepatitis B vaccinated HC. Scatter plot of IL2 release of HepB vaccinated HC vs. anti-HBs titers after stimulation of whole blood with **a** HBsAg alone (n = 87), **b** HBsAg and C3a (n = 80), **c** HBsAg and C5a (n = 74) as well as **d** HBsAg, C3a and C5a (n = 55). Antigen induced cytokine responses were corrected by the negative control values. Continuous line indicates linear regression and Pearson correlation. R^2^: coefficient of determination

The same was true with respect to IL2 (Fig. 7, (a) R^2^ = 0.32 and p < 0.0001 for HBsAg stimulation; (b) R^2^ = 0.37 and p < 0.0001 for HBsAg + C3a stimulation; (c) R^2^ = 0.32 and p < 0.0001 for HBsAg + C5a stimulation; (d) R^2^ = 0.29 and p < 0.0001 for HBsAg + C3a and C5a stimulation).

### Effects of C3a and C5a on T cells and antigen presenting cells

In a first attempt to unravel the mechanisms by which the anaphylatoxins enhance the IFNγ release assay sensitivity, we analyzed the activation state of the T cells and the APCs upon stimulation of whole blood with HBsAg and HBsAg combined with C3a and C5a, respectively. Previous studies demonstrated that the anaphylatoxins C3a and C5a are able to modulate T cell responses by altering the expression levels of for instance costimulatory molecules [11, 12].

Thus, we used a flow cytometry-based approach to analyze the expression levels of costimulatory molecules as well as activation markers on T cells as well as on APCs (Additional file 1: Figure S1). More specifically, with regard to T cells, we measured expression levels of CD28 and CD25. We could not detect any significant changes in the expression levels of CD25 (Additional file 2: Figure S2a) and CD28 (Additional file 2: Figure S2b) on T cells when stimulating whole blood with HBsAg in combination with the anaphylatoxins C3a and C5a. Even when looking at the cytotoxic and the T helper (Th) cell subsets there were no differences (Additional file 3: Figure S3).

With regard to the MHCII^+^ APCs, we analyzed the expression levels of MHCII as well as of the costimulatory molecules CD80 and CD86 using the gating strategy shown in Additional file 4: Figure S4. Interestingly, addition of C3a and C5a did not induce upregulation of CD80 (Additional file 5: Figure S5b), but we found a tendency that it might enhance expression levels of MHCII (Additional file 5: Figure S5a) and CD86 (Additional file 5: Figure S5c) since we detected an upregulation of both molecules in 4 of 5 cases.

## Discussion

Hepatitis B represents a major global health problem with numerous patients developing chronic hepatitis B leading to liver cirrhosis or hepatocellular carcinoma [1]. Therefore it is of major importance to ensure quick diagnosis and subsequent treatment.

Whole blood-based cytokine release assays serve as powerful, easy as well as cost-effective tools to analyze cellular immune responses to various pathogens including *Mycobacterium tuberculosis* and HBV. Beyond that, our data demonstrate that this method is well suited to rapidly assess the HBV vaccination status of patients. In this context, HBsAg, as a major component of the available prophylactic HBV vaccine, is up to now the antigen of interest with regard to vaccination status analysis and we could show HBsAg-induced cytokine release in blood of HepB vaccinated donors.

Unfortunately, low frequencies of antigen-specific memory T cells in peripheral blood negatively affect cytokine release assay sensitivity and specificity and therefore it is important to find solutions to solve this problem. Meanwhile, it is well accepted that innate signals mediated via TLRs are able to promote antigen-induced cytokine release and may thereby help to optimize assay sensitivity. Previously published data from our group show that a CMV specific cytokine release assay could be optimized when whole blood is stimulated with synthetic CMV peptides and TLR2 or TLR3 agonists [4]. Further, the TLR2 agonists lipoteichoic acid (LTA) and peptidoglycan (PGN) increase IFNγ synthesis specifically during CEFT antigen challenge [5]. Additionally, with respect to an HBsAg-specific cytokine release assay we could recently demonstrate that CpG oligonucleotides (CpG ODN) as TLR9 agonists enhance IFNγ release assay sensitivity [6].

The data depicted here demonstrate that not only TLR agonists, but also the complement factors C3a and C5a modulate HBsAg-specific cellular immune responses and affect assay sensitivity. We show that both, C3a and C5a, increase IFNγ release assay sensitivity, C3a by around 7%, C5a by more than 20% and the combination of both complement components even by almost 30%.

The interesting fact that C3a positively affects assay sensitivity without significantly altering IFNγ production, in general, suggests that this anaphylatoxin specifically modulates cytokine responses in HBV-vaccinated HC.

The diagnostic sensitivity of the IL2 release assay was with 84.8% better than the one of the IFNγ release assay and could not be optimized by a combination of HBsAg with the complement factors C3a and C5a.

As already mentioned, our HBV-specific IFNγ release assay reached a diagnostic sensitivity of up to 78.9% at a diagnostic specificity of 90% when the complement factors were used for stimulation of whole blood. In comparison, the same assay only reached a diagnostic sensitivity of 76% when CpG ODN was applied as published recently [6]. The percentage increase was with up to 29.8% much higher for the complement factors than for CpG ODN with only 15%.

Since complement receptor pathways have been shown to intersect with TLR pathways [7, 13] it is entirely possible that TLR agonists as CpG ODN and the anaphylatoxins C3a and C5a synergize to modulate HBsAg-driven cytokine secretion. Thus, it is worth analyzing potential synergistic effects with regard to benefits for the HBV-specific cytokine release assay.

In accordance with previously published data from our group [6] we could show a clear correlation between the anti-HBs antibody titer and the HBsAg-mediated secretion of IL-2 in whole blood in line with the current study. Moreover, we could also show such a correlation with regard to IFNγ for the first time what might be explained by the slightly larger size of the study group (59 instead of 51).

Apart from that, in a next step all positive effects of innate stimuli (TLR agonists, complement factors) on the sensitivity of the HBV-specific cytokine release assay that have been shown in line with vaccination studies with HC have to be validated in studies with hepatitis B patients. Clinical trials with therapeutic hepatitis B vaccines rely on a robust cellular immune response which is in contrast to the general hyporesponsive and immune exhausted state of HBV specific T cells in chronic hepatitis B patients [14, 15]. Antibody titers do not mirror cellular immunity, e.g. of cytotoxic T cells, and HBV antigen levels are not helpful, either. Thus, we propose our protocol as an additional easy-to-use, cost efficient and robust tool for therapeutic hepatitis B vaccination studies.

Our first attempt to analyze the mechanisms by which the anaphylatoxins enhance the IFNγ release assay sensitivity suggests that an additional stimulation of whole blood with the C3a and C5a does not alter the T cell activation state since we found neither a regulation of the CD25 nor of the CD28 expression levels. In contrast, there might be an effect of the anaphylatoxins on the APCs. MHCII and CD86 expression levels increased in 4 of 5 cases upon addition of C3a and C5a. Anyhow, we have to consider the relatively small size of the study group (n = 5) and the limited amount of analyzed surface molecules.

Apart from that, there are different factors making an in depth analysis of the regulatory mechanisms a challenging task. First, the anaphylatoxins C3a and C5a exert numerous different effector functions targeting a broad spectrum of immune, but also non-immune cells [16]. Thus, it is hard to say if the anaphylatoxins act directly or indirectly on our cells of interest. Further, control mechanisms have evolved to regulate the activity of these powerful bioactive molecules. More specifically, carboxypeptidases, present for instance in the serum, degrade C3a and C5a by cleaving off their C-terminal arginine (Arg) residue, generating so-called C3adesArg and C5adesArg anaphylatoxin fragments [17, 18]. Since our test system is based on stimulation of whole blood, a potential degradation of the added anaphylatoxins has to be taken into consideration. While C3adesArg does not retain any inflammatory function, C5adesArg still shows pro-inflammatory potential [19, 20]. Anyhow, degradation of C5a alters the affinity of the molecule for its two different receptors [21]. Apart from the G-protein coupled receptors C5aR1 there is a second receptor for C5a, C5aR2, whose functions are still controversially discussed [22]. Taken together, the mentioned points emphasize that a separate study is needed to shed light on the mechanisms by which the anaphylatoxins enhance the IFNγ release assay sensitivity. We aim at performing an according study in future.

## Conclusions

Complement factors C3a and C5a massively increase the diagnostic quality of HBV IGRAs. Innate signals mediated via complement pathways contribute to HBV-specific cellular immune responses. The massively improved diagnostic sensitivity of the IFNγ release assay after addition of C3a and C5a demonstrates that these effects render whole blood-based cytokine release assays even more potent as screening tools in HBV immunology and HBV vaccination studies.

## Materials and methods

### Material

The recall antigen pool CEFT was purchased from JPT, Germany (#PM-CEFT) and stored at − 20 °C after reconstitution (25 mg/ml in DMSO). CEFT antigen pool consisted of antigenic peptides from human Cytomegalovirus (HHV-5; CMV), Epstein–Barr virus (HHV-4; EBV), Influenza A and *Clostridium tetani*. This positive control pool contained 27 peptides selected from defined HLA class I and II-restricted T-cell epitopes. Considering the high vaccination frequency against Influenza and *C. tetani* and the high prevalence of CMV and EBV in the general population in Germany recall antigen responses were expected for all patient samples. *Staphylococcus aureus* enterotoxin B (SEB) was purchased from Sigma Aldrich GmbH, Germany (#S4881) and stored at − 20 °C (1 mg/ml in sterile, endotoxin-free H_2_O). Synthetic HBV peptide libraries of HBcAg and HBsAg were purchased from JPT, Germany (#PM-HBV-CP and #PM-HBV-lEP, respectively) and stored at − 20 °C after reconstitution (50 mg/ml in DMSO). Synthetic HBcAg represented a mix of 44 peptides (15 amino acids each, 11 aa overlap, peptide scan 15/11) comprising the whole amino acid sequence of HBcAg, the 21.5 kDa capsid protein of HBV (genotype A2 subtype adw2, *UniProt: P0C693*). Synthetic HBsAg represented a mix of 98 peptides (15 amino acids each, 11 aa overlap, peptide scan 15/11) comprising the whole amino acid sequence of HBsAg, the 43.7 kDa surface protein of HBV (genotype A2 subtype adw2, *UniProt: P17101*). DMSO was obtained from Th. Geyer, Germany (#RO/A9941/000100). Glucose was provided by Carl Roth, Germany (#HN06.1). Human C3a and human recombinant C5a were purchased from Hycult Biotech, Germany (#HC2126, #HC2101). C3a was reconstituted in ddH_2_O (0.5 mg/ml) and stored at − 80 °C whereas C5a was reconstituted in ddH_2_O (0.15 mg/ml) and stored at − 20 °C. ELISA MAX Deluxe Sets for IFNγ (#430106) and IL2 (#431806) were obtained from BioLegend, Germany. PolyHRP80 streptavidin conjugate was purchased from SDT Reagents, Germany (#SP80C).

The following antibodies were used in line with this study: anti-CD3 BV605 (OKT3), anti-CD4 FITC (A161A1), anti-CD8a PE (HIT8a), anti-CD25 BV421 (BC96), anti-CD28 APC (CD28.2), anti-HLA-A, B, C BV605 (W6/32), anti-HLA-DR APC (L243), anti-CD80 BV711 (2D10), anti-CD86 PeCy7 (IT2.2) and Human TruStain FcX™ (Fc Receptor Blocking Solution). All were purchased from BioLegend. Erythrocytes were eliminated using Red Blood Cell Lysis Buffer (BioLegend) and Live/Dead staining was performed using Zombie NIR™ Fixable Viability Kit (BioLegend). DPBS with 1% FBS (Thermo Fisher Scientific) and 0.09% sodium azide (Sigma-Aldrich) was used as FACS staining buffer.

### Blood donor selection

Eighty-seven healthy donors of the blood transfusion service at the University Medical Center Hamburg-Eppendorf were enrolled anonymously in the study (Table 1), which was approved by the Ethics Committee of the Hamburg Chamber of Physicians (WF-051/12). Clinical data such as gender, age, and HBV vaccination status were provided.

### Cytokine release assay in whole blood ex vivo

Venous blood from healthy donors was collected in sterile 7.5 ml Lithium Heparin Monovettes (Sarstedt, Germany). 0.5 ml of whole blood were then transferred to sterile, pyrogen-free 2 ml tubes (Sarstedt, Germany) and stimulated with HBV antigens (HBsAg 50 µg/ml). Samples stimulated with 0.9% (w/v) NaCl solution served as negative control whereas SEB (1 µg/ml) and CEFT (50 µg/ml) stimulated samples served as positive controls. Glucose (2 mg/ml final concentration; pre-diluted in sterile 0.9% (w/v) NaCl solution) was added to each tube to further enhance cytokine secretion as described previously [3]. Following titration, the complement factor C3a was used at a final concentration of 0.1 µg/ml whereas C5a was used at a final concentration of 0.75 µg/ml. All tubes were incubated at 37 °C for 24 h. Upon centrifugation plasma supernatants were aspirated, stabilized with 0.045% (w/v) NaN_3_ and stored at − 20 °C until cytokine measurement by ELISA. The amount of biomaterial per donor was not always sufficient to perform all stimulations which is why for some donors conditions had to be left out. This explains variations with regard to the group size (n).

### IFNγ and IL2 ELISA

IFNγ and IL2 concentrations in human plasma were determined using ELISA MAX Deluxe Sets (BioLegend) following the manufacturer’s protocol. Anyhow, in order to increase assay sensitivity the manufacturer’s Avidin-horseradish peroxidase conjugate was substituted by a PolyHRP80 streptavidin–horseradish–peroxidase conjugate.

Samples were initially diluted as follows: negative control (NaCl) 1/5, 1st positive control (SEB) 1/2500 for IFNγ and 1/500 for IL2, 2nd positive control (CEFT) 1/50 for IFNγ and 1/25 for IL2, test samples (HBcAg and HBsAg) 1/5. If a test sample’s absorbance value fell outside the standard curve range, these samples were subsequently retested with a tenfold higher or lower dilution, respectively. A seven-point standard curve (1–64 pg/ml) was used for quantitation. All samples were measured in duplicates with a MAX 002 plate reader (Dynex Technologies) followed by data analysis using Microsoft Excel software.

### Anti-HBsAg ECLIA

HBsAg-specific antibodies were quantified in plasma samples using the Elecsys^®^ AntiHBs-Kit (Roche) following the manufacturer’s protocol. The measurements were performed and the according data provided by the Institute for Laboratory Medicine and Microbiology at the University Hospital Brandenburg.

### Flow cytometry

Phenotypic characterization of the whole blood cells upon stimulation was performed using a LSR Fortessa cytometer (BD). For flow cytometric analysis blood was stimulated as described above. Subsequently, red blood cells were lysed by incubating whole blood with Red Blood Cell Lysis Buffer for 20 min at RT. For live/dead discrimination cells were then washed with DPBS (350 g, 5 min), re-suspended in DPBS (1 × 10^7^/ml) and stained with Zombie NIR™ dye for 20 min at RT. Cells were washed with staining buffer (350 g, 5 min) and incubated in staining buffer with Human TruStain FcX™ for 10 min at RT. Subsequently, cells were stained with different sets of antibodies. Anti-CD3 BV605, anti-CD4 FITC, anti-CD8a PE, anti-CD25 BV421 and anti-CD28 APC antibodies were used to assess the activation state of T cells whereas APCs were analyzed with anti-HLA-A, B, C BV605, anti-HLA-DR APC, anti-CD80 BV711 and anti-CD86 PeCy7 antibodies. Cells were stained for 30 min at RT, washed and finally re-suspended in fresh staining buffer.

### Data analysis

#### Software

The ELISA data were analyzed using SPSS software (IBM, version 23) and GraphPad Prism software (Graphpad Software Inc., version number 6.04).

#### Statistical analysis

Descriptive statistics, Shapiro–Wilk normality test, Friedman test, receiver operating characteristic (ROC) analysis and Pearson correlation were performed using SPSS Statistics software (IBM, version number 22) and GraphPad Prism software (Graphpad Software Inc., version number 6.04).

## Additional files

**Additional file 1: Figure S1.** Gating strategy for flow cytometric analysis of T cell activation state. Gating strategy used for flow cytometric analysis of T cells upon stimulation of whole blood with HBsAg and HBsAg combined with C3a and C5a, respectively. First, dead cells and cell doublets were excluded. Within the lymphocyte population CD3^+^ T cells were then identified and cytotoxic T cells and T helper cells were discriminated according to the expression of CD8a and CD4. Finally, expression levels of CD25 and CD28 were quantified on the different T cell subsets. Gates were set according to FMO controls. n = 5.

**Additional file 2: Figure S2.** Expression levels of CD25 and CD28 on CD3^+^ T cells. Expression levels of (a) CD25 and (b) CD28 on CD3^+^ T cells upon stimulation of whole blood with HBsAg and HBsAg combined with C3a and C5a. Depicted is the mean fluorescence intensity (MFI). All MFI values were corrected by the according FMO controls. n = 5.

**Additional file 3: Figure S3.** Expression levels of CD25 and CD28 on T cell subsets. Expression levels of the activation marker (a) CD25 and (b) CD28 on CD4^+^ Th cells as well as expression levels of (c) CD25 and (d) CD28 on CD8a^+^ cytotoxic T cells upon stimulation of whole blood with HBsAg and HBsAg combined with C3a and C5a. Depicted is the mean fluorescence intensity (MFI). All MFI values were corrected by the according FMO controls. n = 5.

**Additional file 4: Figure S4.** Gating strategy for flow cytometric analysis of APC activation state. Gating strategy used for flow cytometric analysis of APCs upon stimulation of whole blood with HBsAg and HBsAg combined with C3a and C5a, respectively. First, dead cells and cell doublets were excluded. Within the MHCI^+^ population MCHII^+^ APCs were then identified and analyzed for expression of CD80 and CD86. Gates were set according to FMO controls. n = 5.

**Additional file 5: Figure S5.** Expression levels of MCHII, CD80 and CD86 on APCs. Expression levels of (a) MHCII, (b) CD80 and (c) CD86 on MHCII^+^ APCs upon stimulation of whole blood with HBsAg and HBsAg combined with C3a and C5a. Depicted is the mean fluorescence intensity (MFI). All MFI values are corrected by the according FMO controls. n = 5.
