# Supplementary figures and images for: Complement factors C3a and C5a mimick a proinflammatory microenvironment and increase HBV IGRA sensitivity
Source: J Transl Med. 2019 Jan 3;17:6. doi: 10.1186/s12967-018-1752-8 (PMC6317231; doi:10.1186/s12967-018-1752-8)

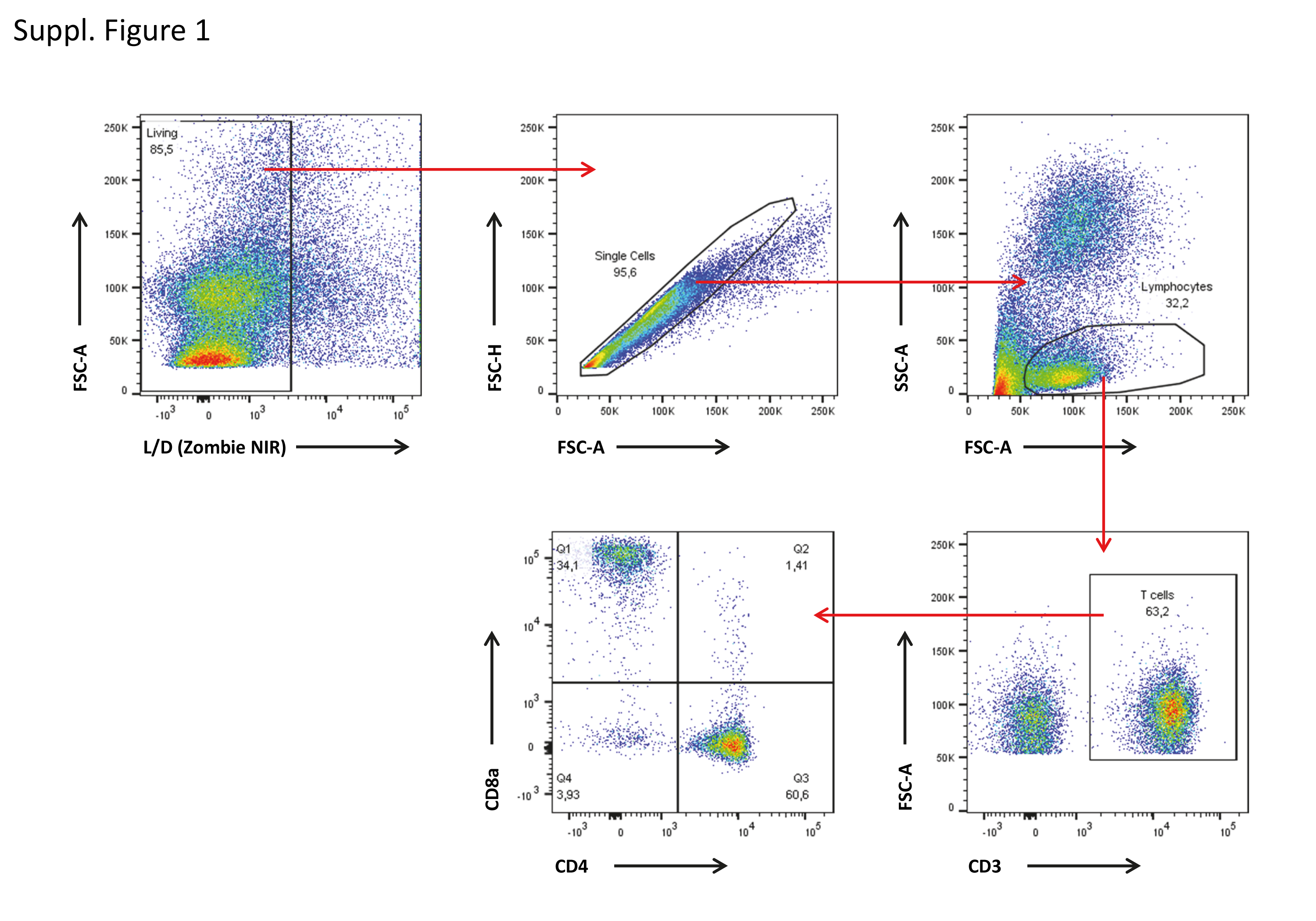

Supplement: Supplementary file 1 — Additional file 1: Figure S1. Gating strategy for flow cytometric analysis of T cell activation state. Gating strategy used for flow cytometric analysis of T cells upon stimulation of whole blood with HBsAg and HBsAg combined with C3a and C5a, respectively. First, dead cells and cell doublets were excluded. Within the lymphocyte population CD3+ T cells were then identified and cytotoxic T cells and T helper cells were discriminated according to the expression of CD8a and CD4. Finally, expression levels of CD25 and CD28 were quantified on the different T cell subsets. Gates were set according to FMO controls. n = 5. [file 12967_2018_1752_MOESM1_ESM.tif]

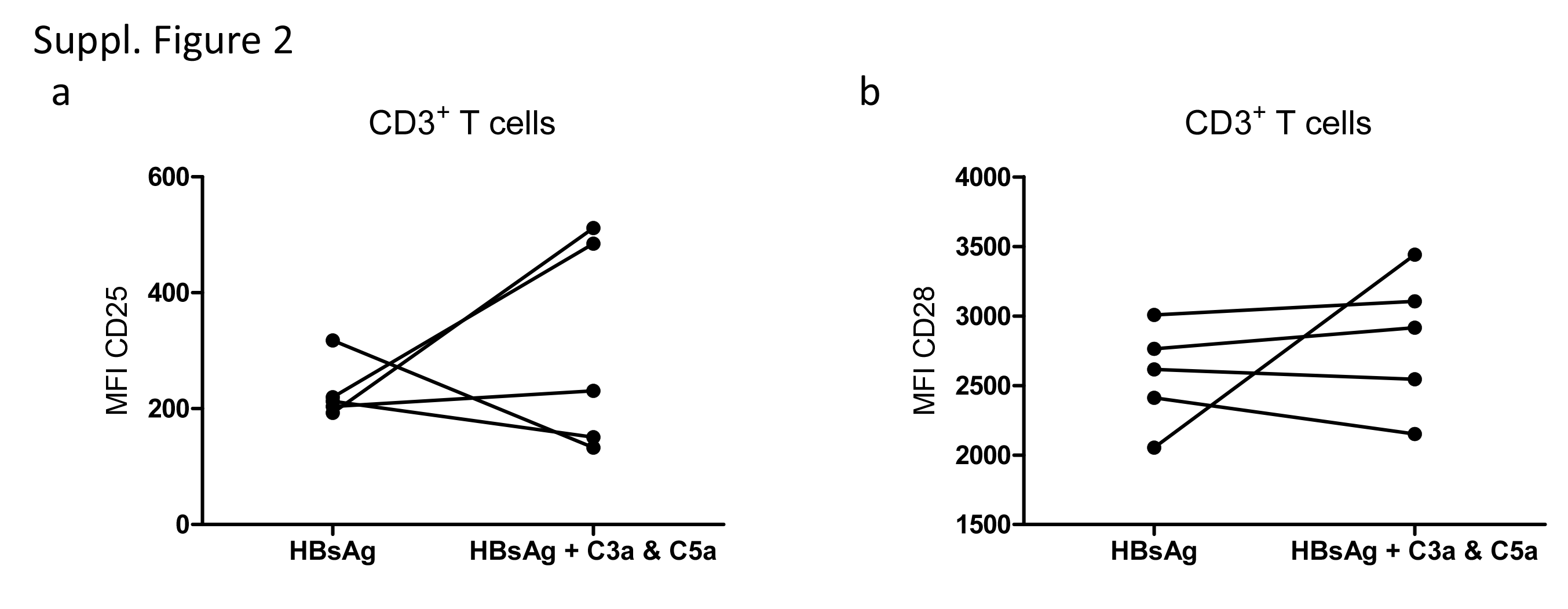

Supplement: Supplementary file 2 — Additional file 2: Figure S2. Expression levels of CD25 and CD28 on CD3+ T cells. Expression levels of (a) CD25 and (b) CD28 on CD3+ T cells upon stimulation of whole blood with HBsAg and HBsAg combined with C3a and C5a. Depicted is the mean fluorescence intensity (MFI). All MFI values were corrected by the according FMO controls. n = 5. [file 12967_2018_1752_MOESM2_ESM.tif]

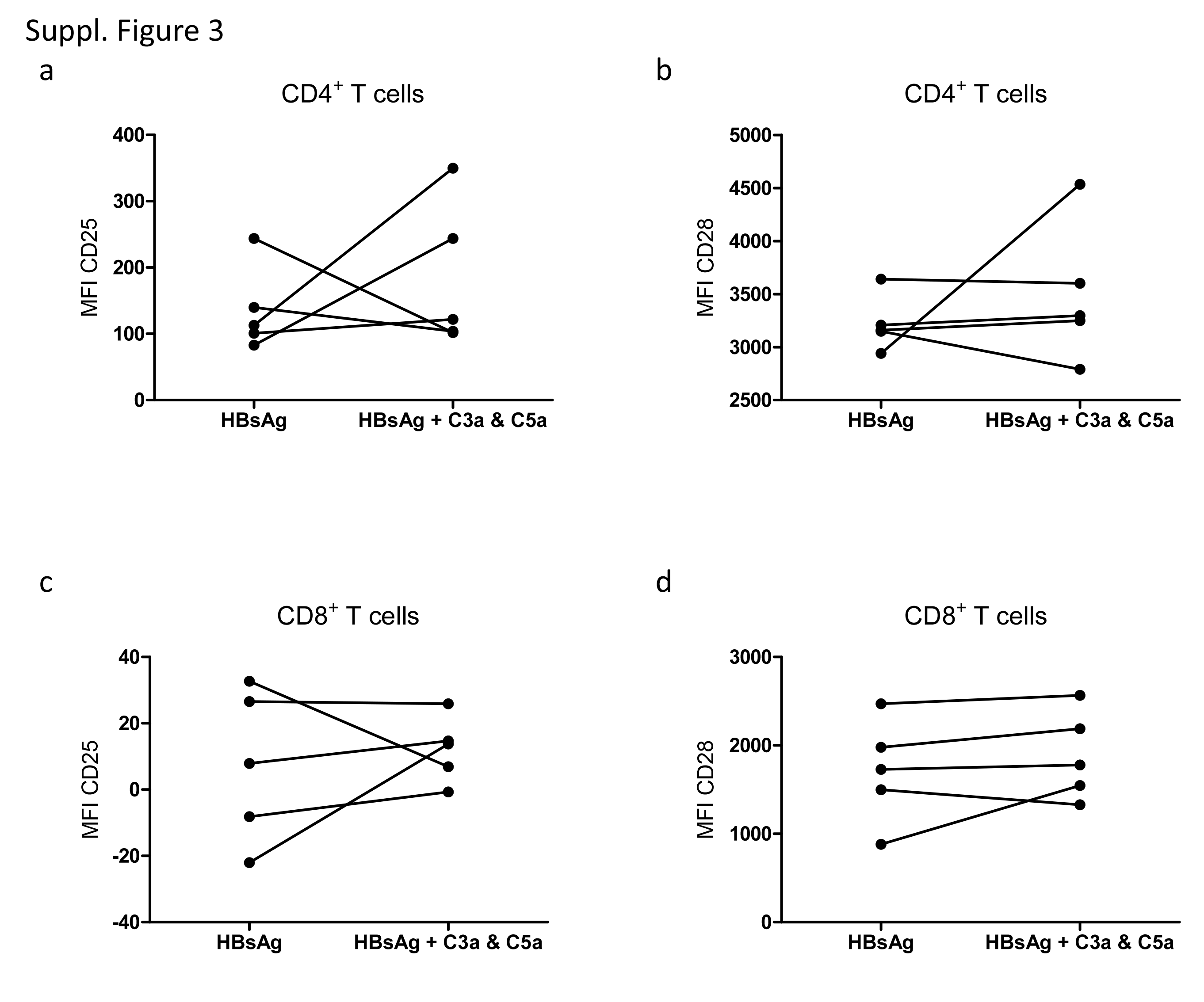

Supplement: Supplementary file 3 — Additional file 3: Figure S3. Expression levels of CD25 and CD28 on T cell subsets. Expression levels of the activation marker (a) CD25 and (b) CD28 on CD4+ Th cells as well as expression levels of (c) CD25 and (d) CD28 on CD8a+ cytotoxic T cells upon stimulation of whole blood with HBsAg and HBsAg combined with C3a and C5a. Depicted is the mean fluorescence intensity (MFI). All MFI values were corrected by the according FMO controls. n = 5. [file 12967_2018_1752_MOESM3_ESM.tif]

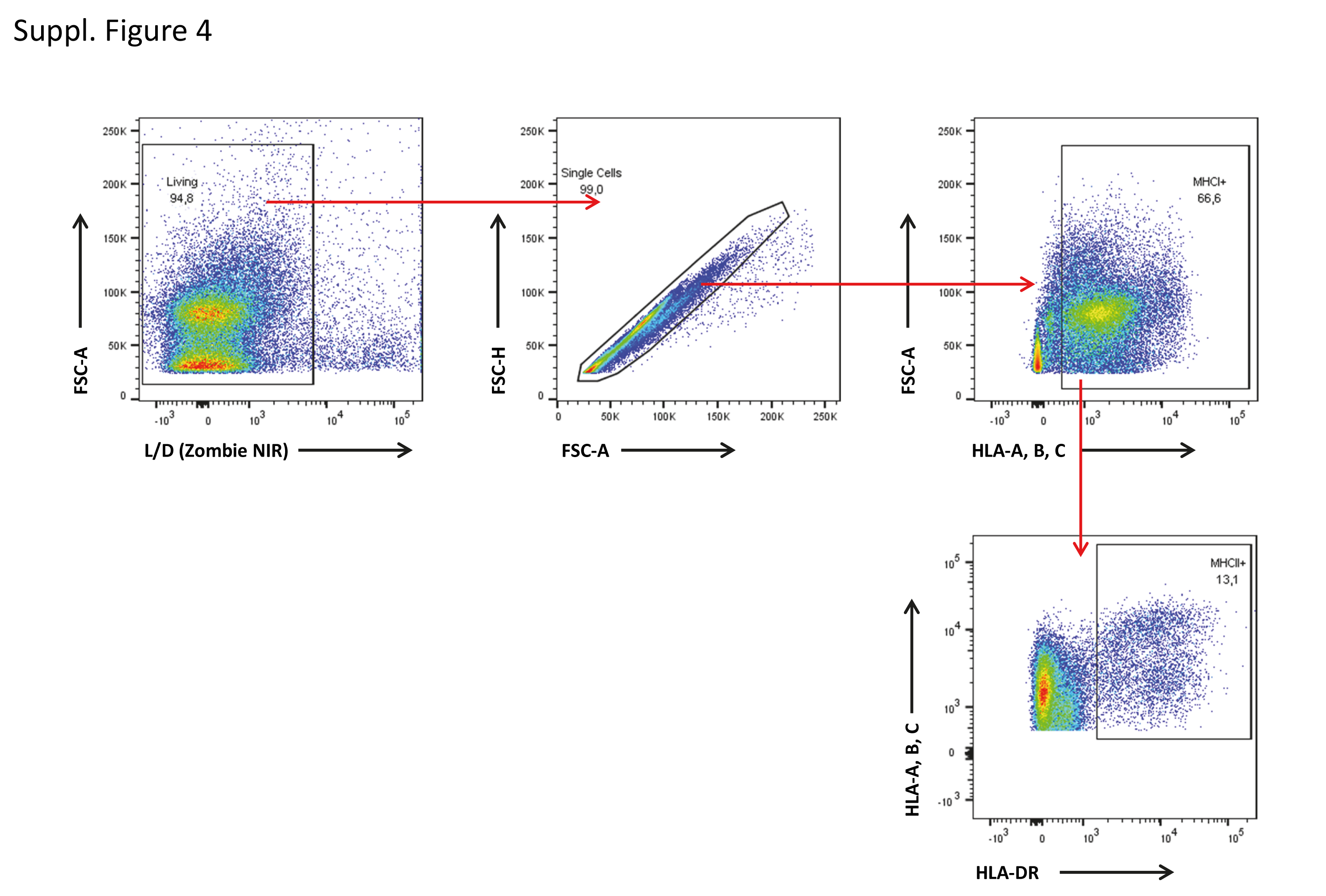

Supplement: Supplementary file 4 — Additional file 4: Figure S4. Gating strategy for flow cytometric analysis of APC activation state. Gating strategy used for flow cytometric analysis of APCs upon stimulation of whole blood with HBsAg and HBsAg combined with C3a and C5a, respectively. First, dead cells and cell doublets were excluded. Within the MHCI+ population MCHII+ APCs were then identified and analyzed for expression of CD80 and CD86. Gates were set according to FMO controls. n = 5. [file 12967_2018_1752_MOESM4_ESM.tif]

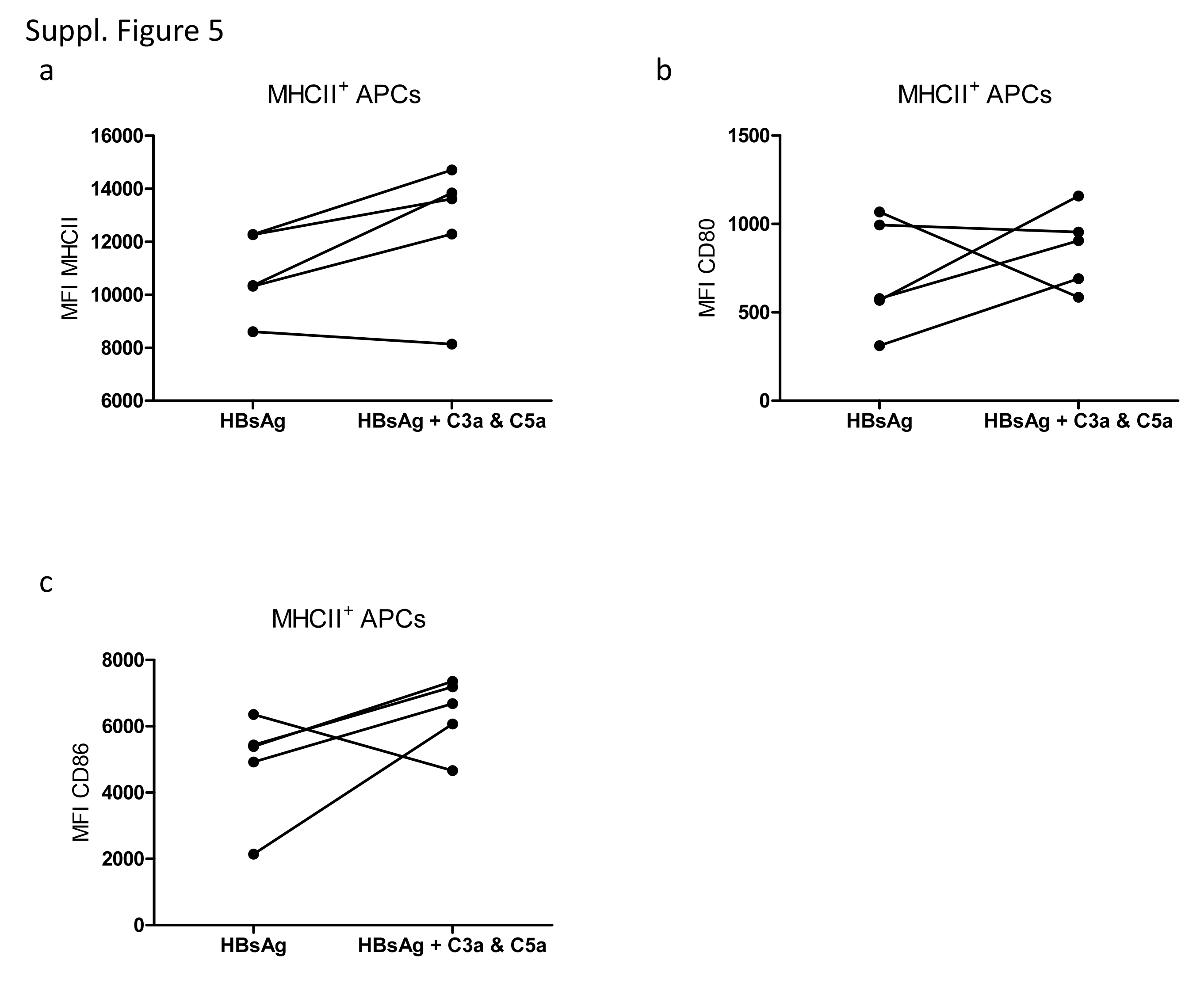

Supplement: Supplementary file 5 — Additional file 5: Figure S5. Expression levels of MCHII, CD80 and CD86 on APCs. Expression levels of (a) MHCII, (b) CD80 and (c) CD86 on MHCII+ APCs upon stimulation of whole blood with HBsAg and HBsAg combined with C3a and C5a. Depicted is the mean fluorescence intensity (MFI). All MFI values are corrected by the according FMO controls. n = 5. [file 12967_2018_1752_MOESM5_ESM.tif]
